# The “New” Disease in Congo: Lessons Learned Compared to Previous Human and Animal Epidemics

**DOI:** 10.3390/pathogens14020122

**Published:** 2025-01-29

**Authors:** Giovanni Di Guardo

**Affiliations:** General Pathology and Veterinary Pathophysiology, Veterinary Medical Faculty, University of Teramo, 64100 Teramo, Italy; gdiguardo@unite.it; Tel.: +39-347-6317862

The “new” disease outbreak in the Democratic Republic of Congo (DRC), which had already been heavily struck by the “Monkeypox” (MPX) epidemic, has been hitherto responsible for approximately 900 cases and 48 fatalities, mostly among children. Despite coinfection with *Plasmodium falciparum*—the causative agent of malaria, which is endemic in Africa—in 80% of these commonly undernourished patients, the diagnostic suspicions raised by the World Health Organization (WHO) included measles, influenza, acute pneumonia, COVID-19, haemolytic uremic syndrome, and malaria (https://www.who.int/emergencies/disease-outbreak-news/item/2024-DON546, accessed on 14 January 2025). This was followed, on 27 December, by an official declaration made by the WHO, stating that the outbreak was caused by respiratory pathogens such as influenza viruses, rhinoviruses, adenoviruses, and SARS-CoV-2, being additionally complicated by malaria as well as by widespread malnutrition [1].

*Mutatis mutandis*, well before the Human Immunodeficiency Virus (HIV)—the agent responsible for Acquired Immunodeficiency Syndrome (AIDS)—was simultaneously identified in 1983 by Luc Montagnier (in France) and Robert Gallo (in the USA), a fungal pathogen formerly termed *Pneumocystis carinii* and subsequently renamed *P. jirovecii* was deemed responsible, being finally (more than two years later!) regarded as one of the many opportunistic microorganisms causing secondary infections in AIDS-affected and, in general, immunocompromised patients [2]. As a matter of fact, fungal and protozoan agents are frequently responsible for opportunistic infections in humans as well as in animals primarily affected by both viral and non-viral immunodeficiencies. This is the case, for instance, of *Canine Distemper Virus* (CDV)-infected dogs and *Cetacean Morbillivirus* (CeMV)-infected dolphins and whales, in which a *Toxoplasma gondii* coinfection may be commonly found, similar to HIV-infected people [3].

Since *P. falciparum* is a protozoan pathogen, an opportunistic role played by this agent in the aetiology of the “new” disease reported in DRC patients should not be ruled out.

Within such a challenging context, when delving into the fascinating history of human and animal infectious diseases, we find that before the discovery of the zoonotic SARS-CoV betacoronavirus in 2002—on behalf of Carlo Urbani, the Italian physician who then succumbed to SARS—*Chlamydia* spp., a bacterial pathogen, was deemed to be the cause of SARS [4]. Likewise, before *Phocine Distemper Virus* (PDV, another *Morbillivirus* genus member) was recognised as the primary aetiological agent of the mass mortality outbreaks affecting North Sea common seal (*Phoca vitulina*) and grey seal (*Halichoerus grypus*) populations in 1988 [5], a *Herpesvirus* was deemed to be responsible for the dramatic die-offs [6].

Invertebrate organisms do not seem to represent an “exception to the rule”; for example, the aetiology of the mass mortality events involving, in recent years, noble pen shells (*Pinna nobilis*) in the Mediterranean Sea region had been attributed to both protozoan (*Haplosporidium pinnae*) and bacterial (*Mycobacterium sherrisii*, *Vibrio mediterranei*) pathogens before the primary causative agent (a small RNA virus belonging to *Picornavirales*) was identified [7].

In conclusion, the discovery of the primary cause(s) of any new human and animal infectious (and non-infectious) disease conditions appear to be preceded, almost unavoidably, by “errors” made by the scientific community before the “true” aetiology becomes unveiled, with this challenging process being made much less complicated and far more charming by multidisciplinary research efforts constantly inspired by the “One Health” principle.

*Historia Magistra Vitae*!
